# Eukaryotic translation initiation factor 5A2 (eIF5A2) regulates chemoresistance in colorectal cancer through epithelial mesenchymal transition

**DOI:** 10.1186/s12935-015-0250-9

**Published:** 2015-11-17

**Authors:** Ying Bao, Yongliang Lu, Xiang Wang, Wenming Feng, Xinrong Sun, Huihui Guo, Chengwu Tang, Xiaojing Zhang, Qilin Shi, Hongbin Yu

**Affiliations:** First Affiliated Hospital, Huzhou Teachers College, The First People’s Hospital of Huzhou, 158 Guangchanghou Road, 313000 Huzhou, China; Huzhou Teachers College of Medicine, 313000 Huzhou, China

**Keywords:** Colorectal cancer, Chemoresistance, eIF5A2, Epithelial mesenchymal transition

## Abstract

**Background:**

Chemoresistance is a major obstacle to successful chemotherapy for colorectal cancer. Eukaryotic translation initiation factor 5A2 (eIF5A2), one of the two isoforms in the eIF5A family, has been reported to be a new oncogene in many types of human cancer. In the present study, we aimed to investigate whether eIF5A2 was involved in the chemoresistance to doxorubicin in colorectal cancer.

**Methods:**

Cell viability was measured by CCK-8 assay with or without doxorubicin treatment. Protein expression was detected by western blot. Tumor cells were transfected with eIF5A2 siRNA or plasmid encoding eIF5A2 to down- or up regulate the expression of eIF5A2.

**Results:**

We found that eIF5A2-negtive colon cancer cells (HCT116 and HT29) were more sensitive to doxorubicin compare with the eIF5A2-positive cells (LOVO and SW480). Downregulation of eIF5A2 in LOVO and SW480 cells enhanced the chemosensitivity to doxorubicin. On the contrary, overexpression of eIF5A2 reduced doxorubicin sensitivity in colon cancer cells. In addition, eIF5A2 knockdown increased the protein level of E-cadherin and reduced vimentin expression in LOVO and SW480 cells. Meanwhile, upregulation of eIF5A2 potentiated epithelial mesenchymal transition (EMT) in colon cancer cells. Moreover, blockade of EMT with Twist siRNA abolished eIF5A2-regulated chemoresistance in colon cancer cells.

**Conclusion:**

Our present study demonstrated that eIF5A2 promoted the chemoresistance to doxorubicin via regulation of EMT in colon cancer cells. Therefore, eIF5A2 inhibition may be a new potential strategy for the reversal of drug resistance in colorectal cancer therapy.

## Background

Colorectal cancer is the second most common cancer in the United States, and its incidence has been increasing in developing countries [1, 2]. It is estimated that over 1 million new cases are diagnosed each year worldwide, and approximately 50 % of these patients die of colorectal cancer [3]. Currently, surgical resection is the optimal treatment for colorectal cancer, and chemotherapy serves as one of the important adjuvant therapies for its treatment [4]. However, the development of acquired drug resistance to conventional chemotherapeutics has become a major obstacle in colorectal cancer treatment [5, 6]. Such limitation highlights the imperative need for identifying novel treatment strategies which may help overcome drug resistance and enhance tumor cell response to anti-cancer drugs.

It is generally believed that carcinogenesis and development of colorectal cancer comprises a series of complicated processes regulated by aberrantly protein expression and alterations of morphological features during malignant progression [7–9]. The term epithelial-mesenchymal transition (EMT) refers to the complicated progress in which tumor cell loses epithelial properties and gains mesenchymal morphology with capacity for metastasis [10, 11]. EMT is involved in wound healing, stem cell behaviour, development, and contributes to cancer progression [12–14]. Emerging evidence suggests that EMT also plays a critical role in the regulation of chemoresistance properties of cancer cells [15, 16]. Eukaryotic translation initiation factor 5A2 (eIF5A2) mainly acts as an elongation factor during mRNA translation step. It has been identified as an oncogene in ovarian cancer, suggesting that aberrant expression of eIF5A2 may be responsible for the malignant behavior of cancer cells [17–19]. However, the relationship of eIF5A2 and drug resistance in colorectal cancer has never been explored. Hence, the present study aimed to investigate the biological role of eIF5A2 in colorectal cancer chemoresistance.

## Results

### Different doxorubicin sensitivity in colon cancer cells

**Fig. 1:**
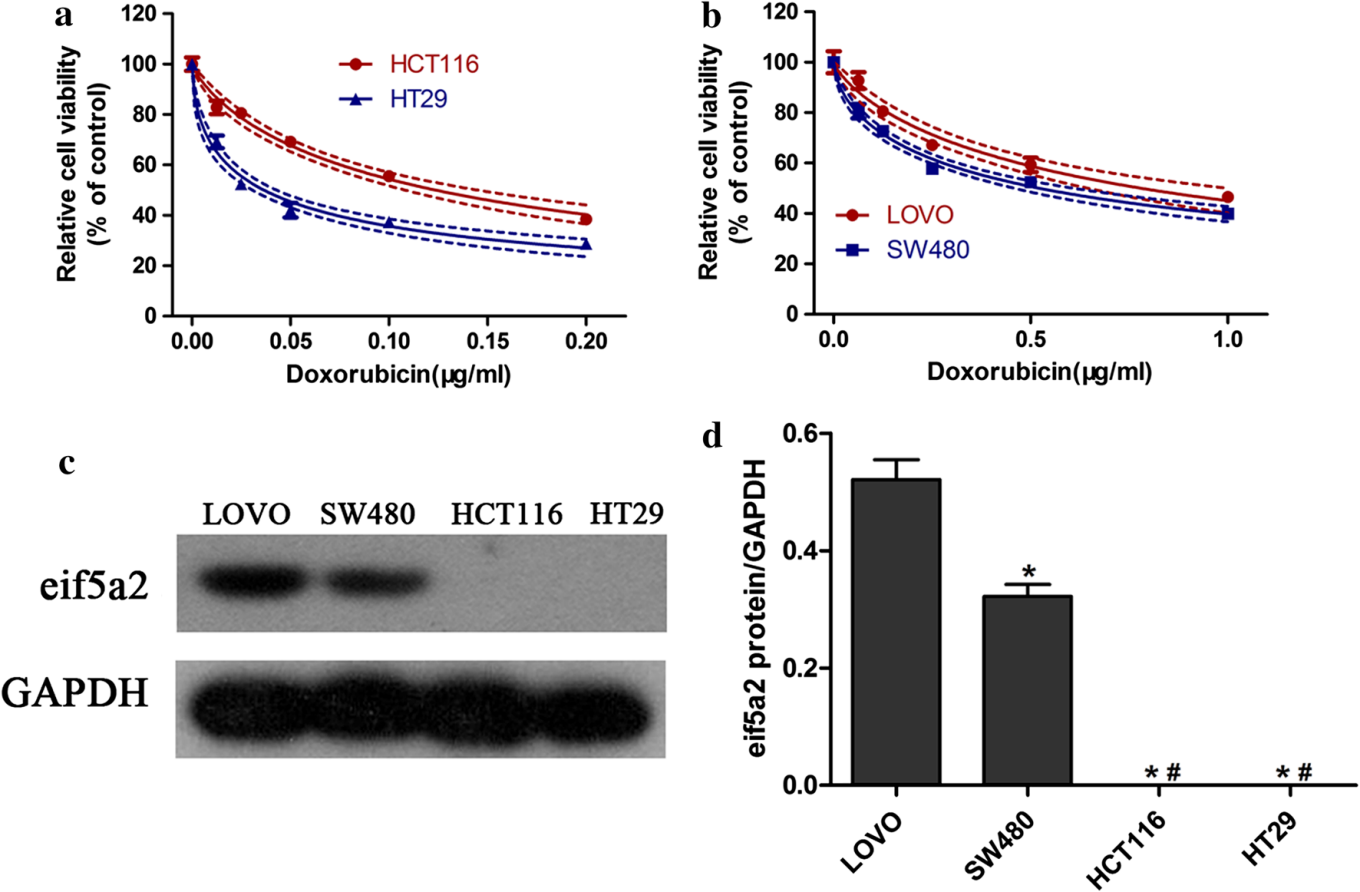
Different doxorubicin sensitivity in colon cancer cells. Four human colon cancer cell lines including HCT116, HT29 (**a**), and LOVO, SW480 (**b**) were incubated with doxorubicin for 48 h. Cell viability was measured using CCK-8 method. Western blot was performed to determine eIF5A2 expression in HCT116, HT29, LOVO and SW480 cells (**c**). *P < 0.05, compared with LOVO; ^#^P < 0.05, compared with SW480

Firstly, CCK-8 assay was performed to measure the sensitivity of different colon cancer cell lines (HCT116, HT29, LOVO and SW480) to doxorubicin. We found that doxorubicin sensitivity varied among cell lines (Fig. 1a, b). As shown in Table 1, the IC_50_ values were significantly higher in LOVO and SW480 cells (0.7810 and 0.5227 μg/mL, respectively) than in HCT116 and HT29 cells (0.1238 and 0.03659 μg/mL, respectively). Specifically, SW480 cells were more sensitive to doxorubicin compared with LOVO cells (Fig. 1b). Western blot analysis demonstrated that eIF5A2 was expressed in LOVO and SW480 cells but no in HCT116 and HT29 cells (Fig. 1c). Interestingly, we observed the highest expression of eIF5A2 in LOVO cells, which were the most insensitive colon cancer cells to doxorubicin. These results implied that eIF5A2 may be involved in the chemoresistance of colon cancer cells.

**Table 1:** IC_50_ values of doxorubicin in colorectal cancer cell lines

Colon cancer cell lines	IC_50_ (μg/mL)
HCT116	0.1238
HT29	0.03659
LOVO	0.7810
SW480	0.5227

### Downregulation of eIF5A2 sensitized colon cancer cells to doxorubicin

**Fig. 2:**
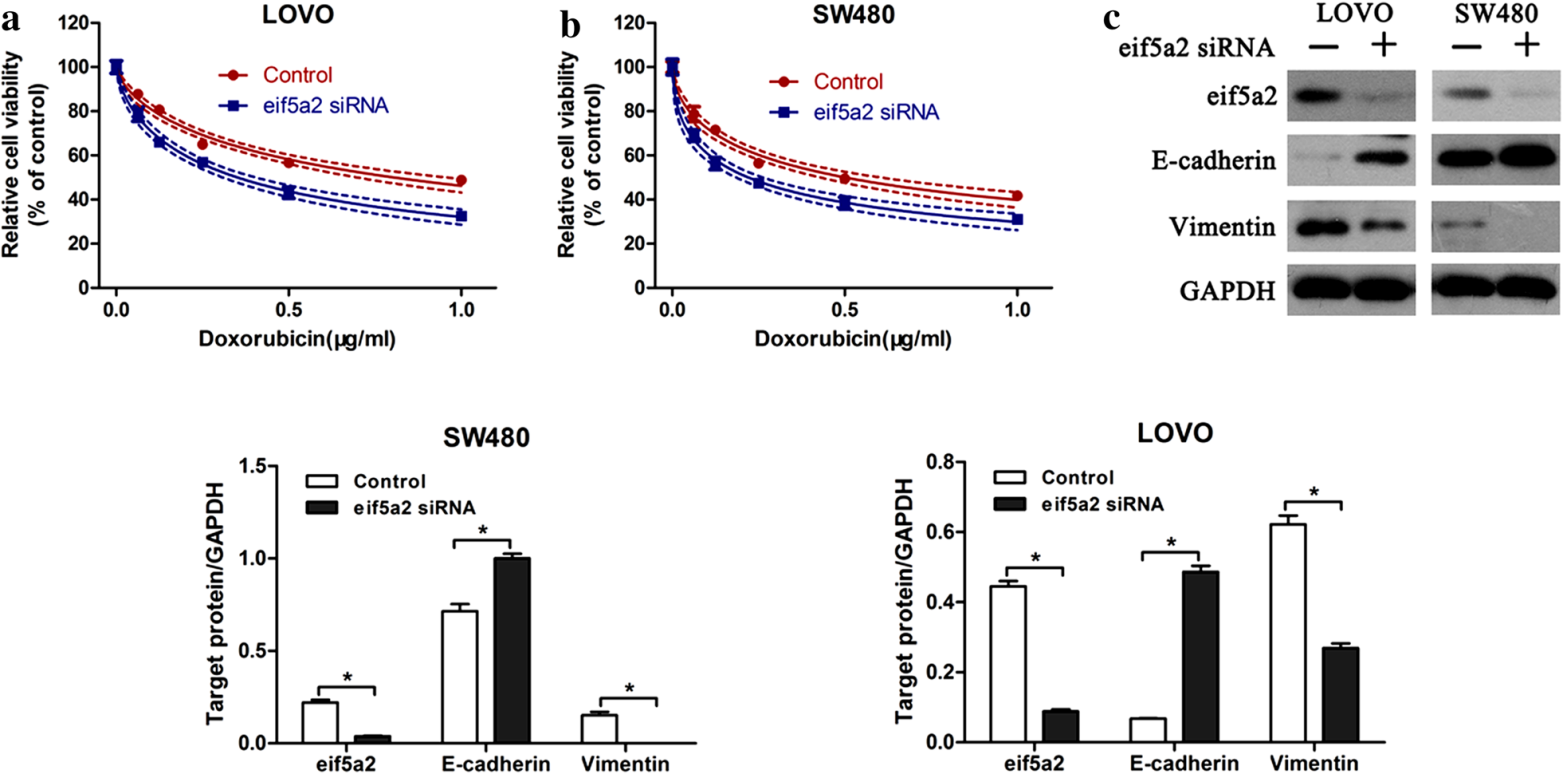
Downregulation of eIF5A2 sensitized colon cancer cells to doxorubicin. CCK-8 assay was performed to measure the cell viability of LOVO (**a**) and SW480 (**b**) after transfection with eIF5A2 siRNA or control siRNA. Western blot analysis of E-cadherin and Vimentin expression in eIF5A2 siRNA or control siRNA transfected colon cancer cells. Relative protein expression in LOVO and SW480 cells was quantified by band density with GAPDH served as control (**c**). *P < 0.05

In order to confirm that eIF5A2 participated in chemoresistance to doxorubicin, eIF5A2 siRNA was transfected into LOVO and SW480 cells. We found that downregulation of eIF5A2 enhanced doxorubicin sensitization in LOVO (Fig. 2a) and SW480 (Fig. 2b) cells. In addition, western blot analysis indicated that eIF5A2 knockdown promoted the expression of E-cadherin and reduced vimentin expression in LOVO and SW480 cells (Fig. 2c). These results demonstrated that downregulation of eIF5A2 prevented EMT and restored doxorubicin sensitivity in colon cancer cells.

### eIF5A2 overexpression reduced doxorubicin sensitivity in colon cancer cells

**Fig. 3:**
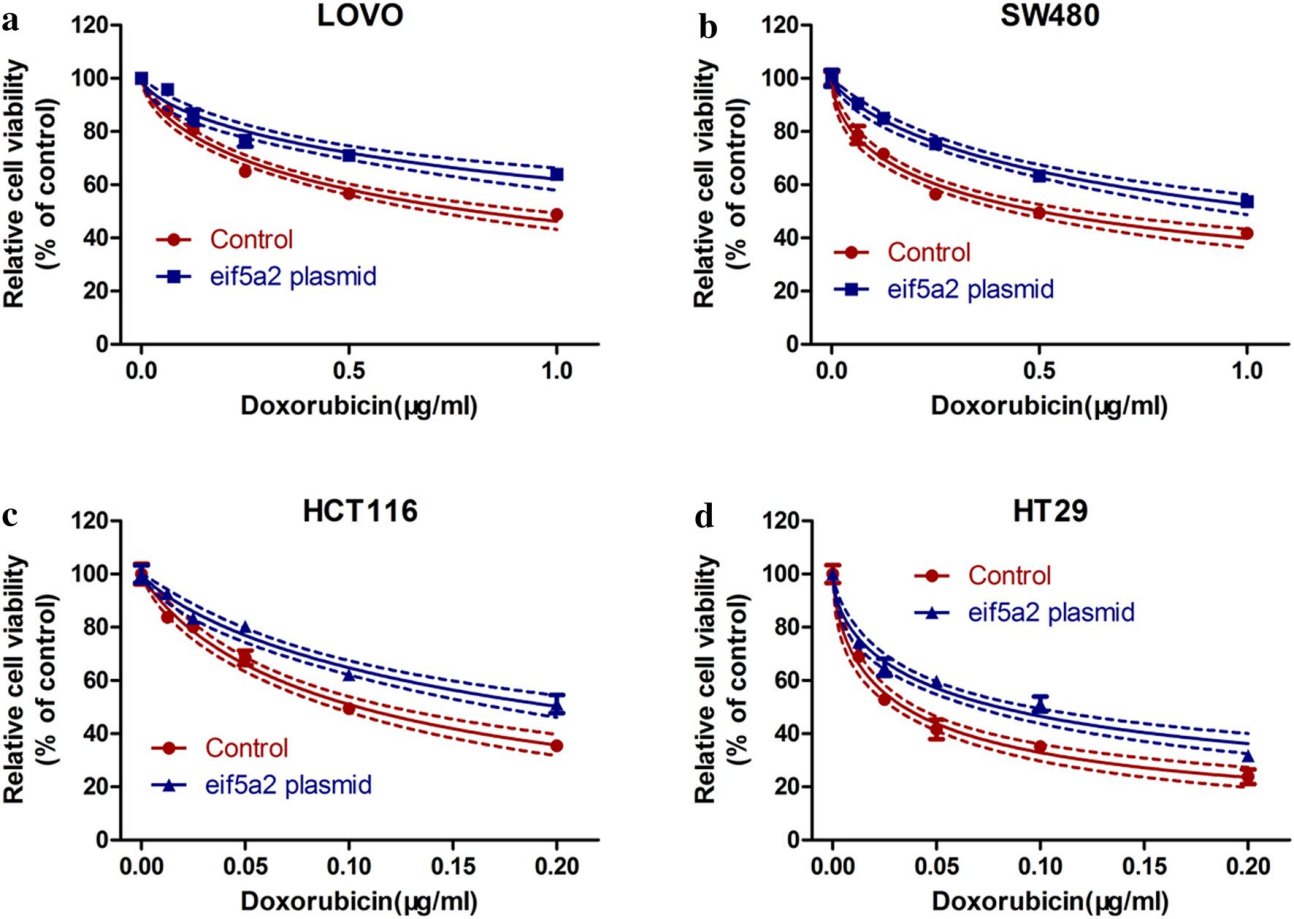
eIF5A2 overexpression reduced doxorubicin sensitivity. eIF5A2 plasmids were transfected into LOVO (**a**), SW480 (**b**), HCT116 (**c**) and HT29 (**d**) cells. 48 h after transfection, CCK-8 assay was performed to measure cell viability in the presence of doxorubicin

Next we transfected colon cancer cell lines with plasmids encoding eIF5A2 to investigate the effect of eIF5A2 overexpression on tumor cells response to drugs. For eIF5A2-positive cells, upregulation of eIF5A2 reduced the sensitivity to doxorubicin in LOVO (Fig. 3a) and SW480 (Fig. 3b) cells. In addition, eIF5A2 overexpression also significantly suppressed the doxorubicin sensitivity in eIF5A2-negative cells, i.e. HCT116 (Fig. 3c) and HT29 (Fig. 3d) cells.

### Effect of eIF5A2 overexpression on tumor cell phenotype

**Fig. 4:**
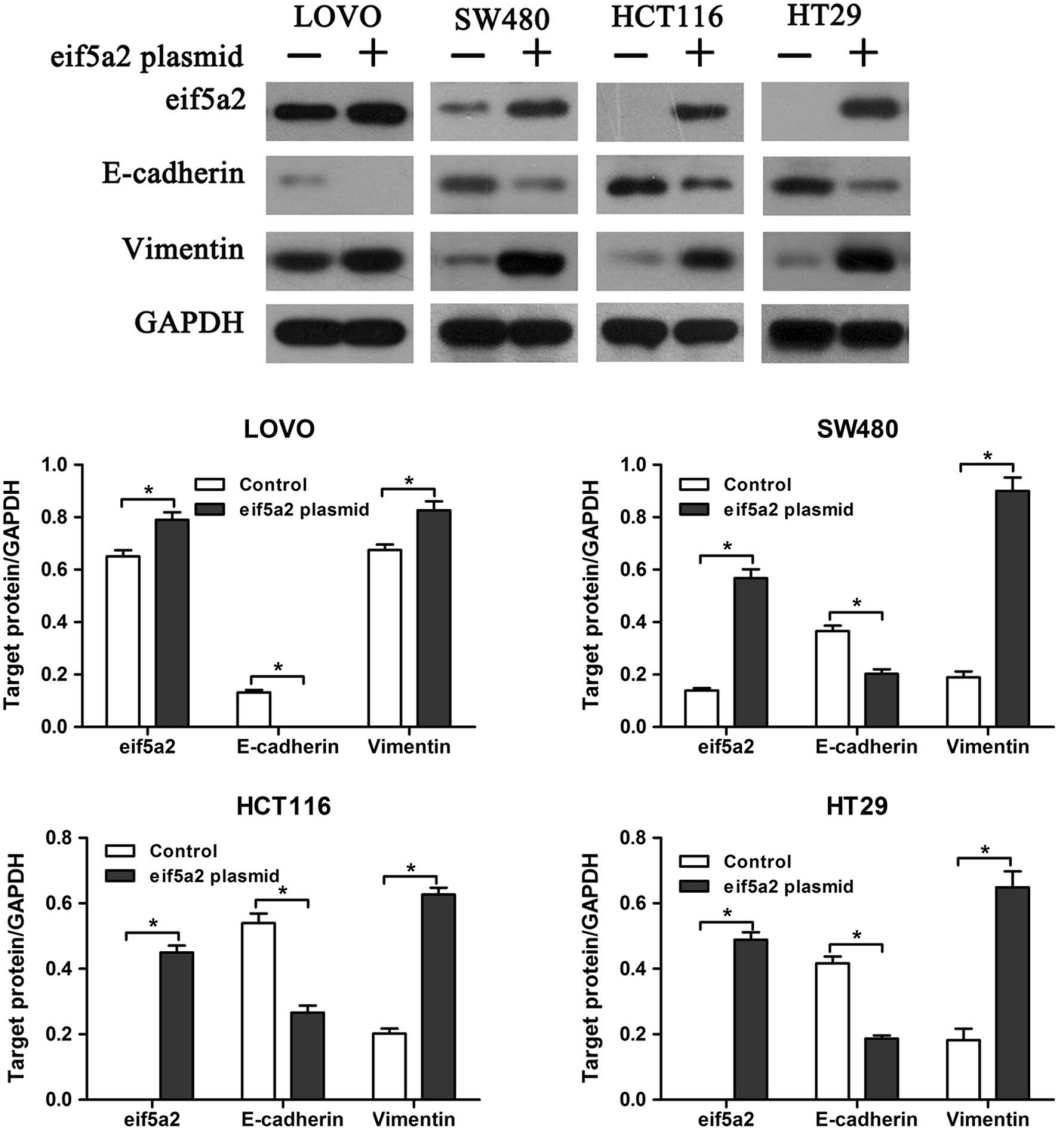
eIF5A2 overexpression altered tumor cell phenotype. Western blot analysis of E-cadherin and Vimentin expression in eIF5A2 siRNA or control siRNA transfected colon cancer cells. Relative protein expression in LOVO, SW480, HCT116 and HT29 cells was quantified by band density with GAPDH served as control. *P < 0.05

**Fig. 5:**
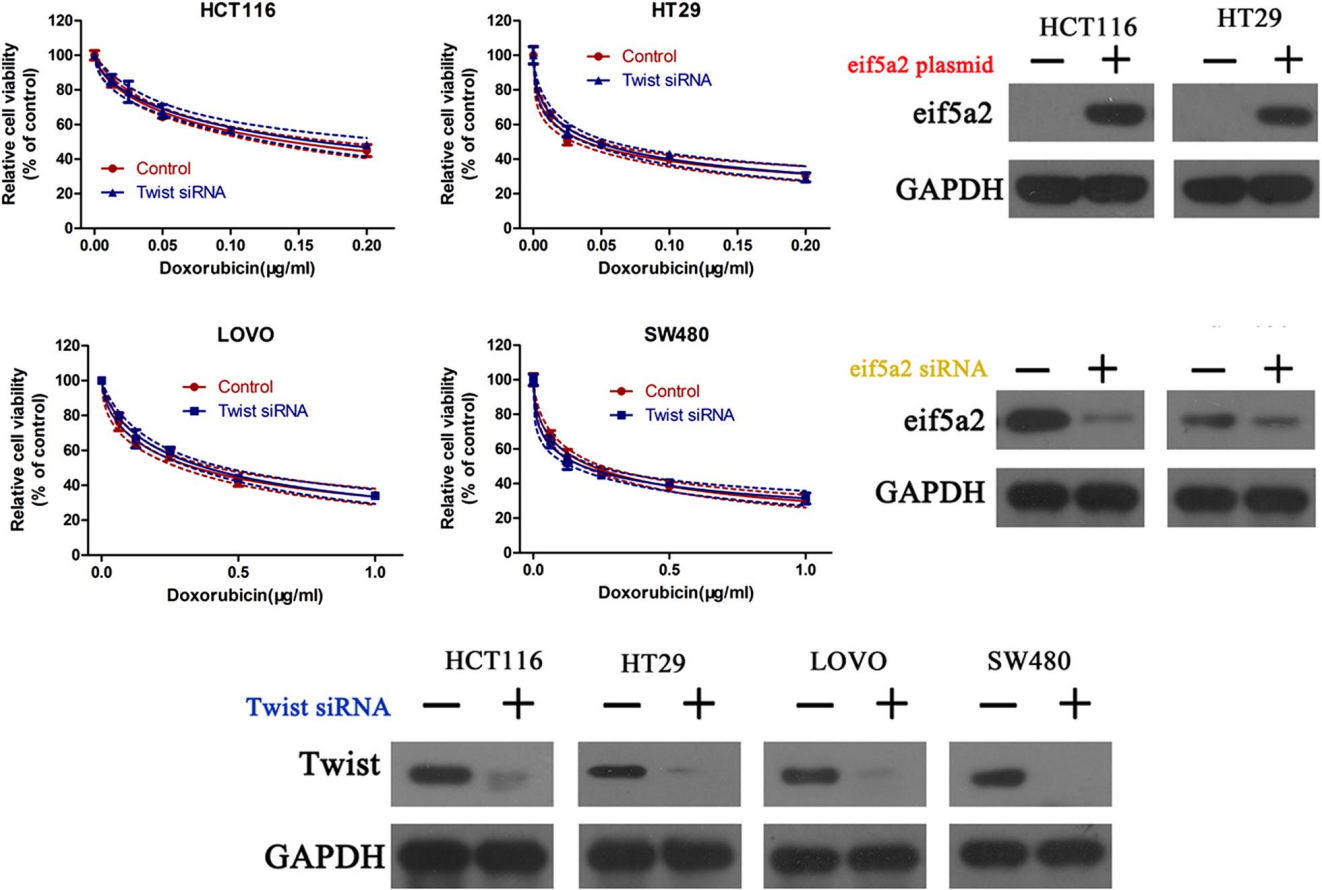
EMT inhibition abolished the role of eIF5A2 on chemoresistance. Twist siRNA and eIF5A2 plasmid were co-transfected into HCT116 and HT29 cells, and Twist siRNA and eIF5A2 siRNA were transfected into LOV0 and SW480 cells. Western blot and CCK-8 assay were performed to measure the protein expression and cell viability, respectively

We further investigated why eIF5A2 overexpression reduced doxorubicin sensitivity in colon cancer cells. We considered the possibility that cell phenotype changes may cause the different responses of colon cancer cell lines to doxorubicin. Not surprisingly, eIF5A2 upregulation significantly reduced the expression of E-cadherin and enhanced vimentin expression in the four colon cancer cell lines (Fig. 4). In addition, Twist siRNA (a critical regulator in EMT) and eIF5A2 plasmid/eIF5A2 siRNA were co-transfected into tumor cells followed by determination of cell viabilities in HCT116, HT29, LOV0 and SW480 cells. Result showed that blockade of EMT with Twist siRNA abolished the regulatory effects of eIF5A2 on cancer cell chemoresistance to doxorubicin, suggesting that EMT was mainly responsible for eIF5A2-mediated chemoresistance (Fig. 5). These findings suggested that regulation of EMT was responsible for eIF5A2-mediated doxorubicin sensitivity in colon cancer cells.

## Discussion

As an important adjuvant treatment, chemotherapy serves as an necessary component of postoperative therapy for colorectal cancer [20]. However, most traditional chemotherapeutic drugs leads to drug resistance and this has become a major obstacle to the triumph of chemotherapy [21]. Therefore, it is imperative to identify novel therapeutic targets which are involved in the acquisition of drug resistance. In this study, we demonstrated that eIF5A2 was associated with the chemoresistance to doxorubicin in colorectal cancer cells.

eIF5A2, an essential component of translation elongation, has been identified to be a novel oncogenic protein in many types of human cancer [17, 18]. Upregulation of eIF5A2 has been reported in many cancers including ovarian cancer, hepatocellular carcinoma, and bladder cancer [22–24]. Both in vitro and in vivo studies suggest that eIF5A2 could promote cancer cell proliferation and increase cancer cell metastasis [22]. Furthermore, it is suggested that eIF5A2 may serve as prognostic biomarker for poor survival of hepatocellular carcinoma patients [25]. In a recent study, eIF5A2 was found to be up-regulated in colorectal cancer patients, and it was suggested to be an independent predictor of shortened survival [26]. Moreover, eIF5A is considered as a potential therapeutic target in many human disorders. In HT-29 and HeLa cells, eIF5A can induce p53-independent apoptosis through mitochondrial pathway [27]. In our study, we found that eIF5A2-negative colon cancer cells were more sensitive to doxorubicin compare with the eIF5A2-positive cells. Thus we hypothesized that eIF5A2 may play an important role in the chemoresistance of colon cancer cells. To test this hypothesis, eIF5A2 siRNA was transfected into LOVO and SW480 cells and we observed enhanced chemosensitivity to doxorubicin. Moreover, eIF5A2 overexpression reduced doxorubicin sensitivity in colon cancer cells. Taken together, these results demonstrated that eIF5A2 promoted the chemoresistance to doxorubicin in colon cancer cells.

It is generally believed that tumorigenesis is a multistep process regulated by aberrantly protein expression and alterations of morphological and molecular features during malignant progression [28]. One such change is the loss of epithelial property and gain of mesenchymal morphology, which suggests the initiation of EMT [29]. Accumulating evidences suggest that EMT plays crucial roles in the acquired chemoresistance in many kinds of cancer, including colorectal cancer [30–32]. Thus, we investigated whether eIF5A2 was involved in regulation of the EMT during the chemoresistance to doxorubicin. Intriguingly, our results demonstrated that overexpression of eIF5A2 significantly decreased the protein level of E-cadherin and increased vimentin expression in HCT116, HT29, LOVO and SW480 cells. On the contrary, downregulation of eIF5A2 reversed the EMT in LOVO and SW480 cells. Furthermore, we found that blockade of EMT with Twist siRNA abolished eIF5A2-regulated chemoresistance. These findings suggested that regulation of EMT was mainly responsible for eIF5A2-mediated doxorubicin sensitivity in colon cancer cells.

In conclusion, our study demonstrated that eIF5A2 promoted the chemoresistance to doxorubicin through regulation of EMT in colon cancer cells. Moreover, specific downregulation of eIF5A2 could reverse the EMT and enhance chemosensitivity to doxorubicin in colon cancer cells. Our study provided a new potential strategy for the reversal of drug resistance in colorectal cancer therapy.

## Methods

### Cell culture

Human colon cancer cell lines HCT116, HT29, LOVO and SW480 were purchase d from the ATCC (Manassas, VA, USA) and cultured in DMEM (Gibco, Carlsbad, CA, USA) supplemented with 10 % FBS and 1 % penicillin⁄streptomycin. All cells were maintained at 37 °C in 5 % CO_2_ incubator.

### Cell viability assay

Tumor cells were seeded onto 96-well plates at 3 × 10^3^ cells⁄well. The medium was replaced with the corresponding serum-free medium for 24 h, then serum-free medium was replaced with complete medium containing the drugs at the indicated doses for 48 h. Then 10 µL⁄well CCK-8 solution (Dojindo, Kumamoto, Japan) was added and incubated with the plates for 3 h, and the absorbance was determined at 450 nm using an MRX II microplate reader (Dynex, Chantilly, VA, USA).

### Transfection

Tumor cells were transfected with eIF5A2 siRNA, eIF5A2 siRNA or plasmid encoding eIF5A2 using Lipofectamine 2000 (Invitrogen, USA) according to the manufacturer’s instruction. The transfection medium (Opti-MEM; Gibco, USA) was replaced with complete medium 12 h after transfection, and the cells were incubated for the indicated times.

### Western blot analysis

Tumor cells were lysed in 50 μL cell lysis buffer (Cell Signaling, Danvers, MA, USA) containing protease inhibitors (Sigma, USA). Whole cell lysates were prepared and fractioned were separated by 10 % SDS-PAGE and proteins were transferred to polyvinylidene difluoride (PVDF) membranes (Millipore, Billerica, MA, USA). The membranes were then incubated with primary antibodies (E-cadherin, Vimentin or eIF5A2, diluted 1:1000; Abcam, Cambridge, USA) at 4 °C overnight. The membranes were washed three times with TBST and then incubated with the appropriate HRP-conjugated secondary antibodies for 1 h at room temperature. Protein expression was detected by chemiluminescence (GE Healthcare, Piscataway, NJ, USA).

### Statistical analysis

Each experiment was performed in triplicate, and repeated at least three times. All the data were presented as mean ± SD and treated for statistics analysis by SPSS program. Comparison between groups was made using ANOVA and statistically significant difference was defined as *P* < 0.05.
